# Detached pyloric gland adenoma of gallbladder: A case report and review of literature

**DOI:** 10.1002/ccr3.9394

**Published:** 2024-10-31

**Authors:** Mohamed H. Khalaf, Lina M. Mohamed, Raed M. Al‐Zoubi, Ahmad Zarour, Mohamed Said Ghali

**Affiliations:** ^1^ Department of Surgery, Acute Care Surgery Hamad Medical Corporation Doha Qatar; ^2^ Surgical Research Section, Department of Surgery Hamad Medical Corporation & Men's Health Doha Qatar; ^3^ Department of Biomedical Sciences QU‐Health, College of Health Sciences, Qatar University Doha Qatar; ^4^ Department of Chemistry Jordan University of Science and Technology Irbid Jordan; ^5^ Weill Cornell Medicine‐Qatar (WCM‐Q) Cornell University Doha Qatar; ^6^ Department of General Surgery Ain Shams University Cairo Egypt

**Keywords:** adenomas, cholecystectomy, detached, gallbladder, pyloric gland adenoma

## Abstract

**Key Clinical Message:**

Vigilant intraoperative inspection is crucial during gallbladder surgery to detect any abnormal tissue including the rare pyloric gland adenomas, which can be easily missed. Thorough examination and removal of unusual lymph nodes or thickened tissues are essential to prevent the risk of malignant transformation and ensure comprehensive patient care.

**Abstract:**

Pyloric gland adenomas are uncommon tumors that can be discovered in various organs such as the stomach, gallbladder, and pancreas. Typically lacking noticeable symptoms, these tumors are categorized into subtypes, with the pyloric variant being the most frequent. The term “intracholecystic papillary‐tubular neoplasms (ICPN)” is used to emphasize growth patterns and associated risks. In a discussed case, a detached pyloric gland adenoma was identified incidentally during a cholecystectomy procedure. A 58‐year‐old obese male presented with upper abdominal pain, nausea, and vomiting. He was diagnosed with early cholecystitis caused by gallstones. Subsequently, during a laparoscopic cholecystectomy, a detached adenoma was discovered. Vigilant screening is crucial, as the identification of gallbladder pyloric gland adenomas is a rare occurrence that might be missed during gallbladder surgery. Any unusual lymph nodes or thickened tissues found in association with the gallbladder ought to be carefully removed, as they could potentially indicate detached gallbladder adenomas with a significant risk of becoming malignant. Failing to consider this possibility may subject the patient to prolonged risks if not meticulously examined.

## 1 INTRODUCTION

Pyloric gland adenomas represent a seldom‐discussed form of neoplasia. They occur in diverse anatomical sites, including the stomach, gallbladder, pancreatic duct, duodenum, and uterine cervix.^1^ Typically, neoplastic polyps of the gallbladder tend to be asymptomatic. They are described in the literature as pyloric gland adenoma, tubulopapillary adenoma, and biliary adenoma.^2^ Histologically, these adenomas are categorized as gastric pyloric gland, gastric foveolar, intestinal, and biliary subtypes, with the pyloric subtype constituting the most prevalent variant (82%).^3^ In surgical literature, patients harboring polyps larger than 1 cm frequently undergo cholecystectomy, which remains the primary treatment approach.^4^ In 2012, Adsay et al. introduced the term “intracholecystic papillary‐tubular neoplasms” (ICPN) to classify gallbladder adenomas based on their growth patterns. Their study highlighted the biliary type as the most frequent (50%), with the pyloric gland subtype (encompassing simple mucinous and complex‐nonmucinous) accounting for 20% of cases. A substantial difference in the risk of invasive behavior was observed among these subtypes, with the biliary subtype displaying a stronger association with invasive carcinoma compared to the pyloric gland subtypes.^2^ Polyps can be adherent to the gall bladder or free within the gall bladder lumen (detached). We present an instance of a detached pyloric gland adenoma encountered in the pericholecystic tissue during a cholecystectomy.

## 2 CASE HISTORY

**FIGURE 1:**
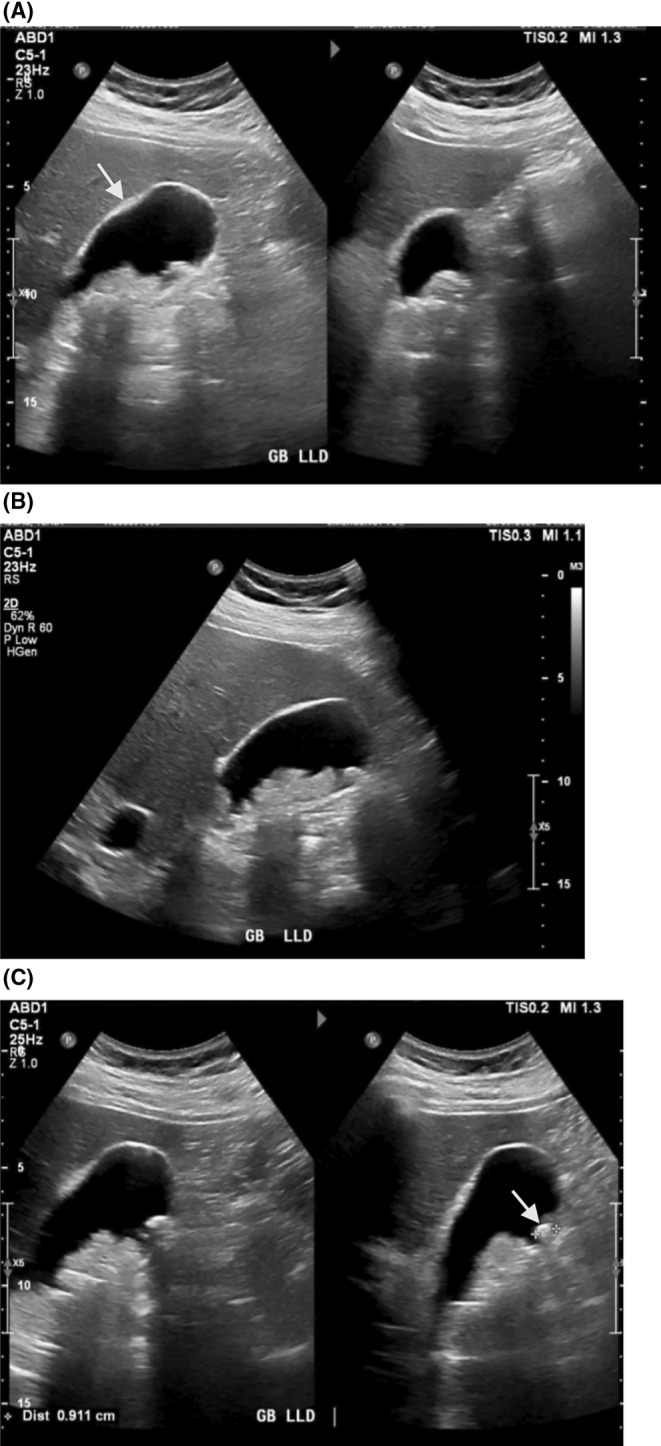
Ultrasound images showing a distended gall bladder (A). No pericholecystic fluid with mild wall thickening of 3.2 mm (arrow). Multiple calculi and internal sludge (B). The largest stone measuring 9 mm (arrow) (C).

A 58‐year‐old male, who is morbidly obese (BMI: 47) and a nonsmoker, presented to the emergency department with a sudden onset of severe sharp right upper abdominal pain lasting for 3 h. The pain was accompanied by feelings of nausea and vomiting. He had experienced similar pain 3 months prior to the presentation. During the examination, his vital signs were within the normal range. Abdominal assessment revealed tenderness and guarding in the right upper quadrant. Laboratory tests showed a slight elevation in liver enzyme levels. An abdominal ultrasound displayed fatty hepatomegaly, splenomegaly, and a distended gallbladder containing multiple calculi and sludge, with the largest calculus measuring 9 mm (Figure 1A–C). The patient was diagnosed with acute caclulous cholecystitis and was taken for a cholecystectomy. An uncomplicated laparoscopic cholecystectomy was successfully performed during the patient's hospitalization.

## 3 METHODS AND RESULTS

Intraoperatively, the gall bladder was found to be acutely inflamed, along with presumed lymphadenopathy. The tissue was delicate and easily damaged, and several small collateral blood vessels were encountered during the procedure. The cholecystectomy was uneventful and hemostasis was achieved. After specimen extraction, the gallbladder was opened, and it contained multiple stones. Both the gall bladder and one excised lymph node were sent for routine histopathological examination. The patient was discharged on the second day after the surgery and had a smooth recovery. The examination of the lymph node specimen showed fragments of tissue that were hemorrhagic and fragile. Upon examination, it was revealed that the presumed lymph node was a detached pyloric gland adenoma, but no lymph node was identified. The gall bladder specimen itself displayed hemorrhagic mucosa that was intermittently disrupted. A hemorrhagic, delicate, polypoidal tissue was also noted in the neck region of the gall bladder, measuring 0.9 cm at its largest point. The final histopathological diagnosis indicated both acute and chronic cholecystitis, as well as a pyloric gland adenoma within the gall bladder.

Despite the surgery, the patient had persistently elevated liver enzymes for over a month. A liver contrast MRI conducted 2 months after the operation revealed the presence of splenomegaly, mild ascites, and early cirrhosis. No lesions were detected in the liver or the gall bladder fossa. Given the intraoperative findings of collateral blood vessels and the subsequent imaging showing splenomegaly, the patient underwent a thorough evaluation to identify any underlying liver disease. It was discovered that the patient had hepatitis C virus, genotype 4, with a high viral load. Consequently, treatment was initiated using sofosbuvir, achieving sustained viral response with undetectable viral load. He is following an exercise regimen and has managed to lose 50 kg after surgery. Follow‐up 3 years after the surgery, he is currently well and under the care of a gastroenterologist.

## 4 DISCUSSION

Gallbladder polyps are identified in approximately 5% of the global population, and their diagnosis has significantly increased due to the widespread use of abdominal ultrasound. These polyps are primarily observed in adults aged between 20 and 94 years. They are either benign or malignant. Benign gallbladder polyps are classified into pseudotumors (cholesterol polyps, inflammatory polyps, cholesterolosis, and hyperplasia), epithelial tumors (adenomas), and mesenchymal tumors (fibroma, lipoma, and hemangioma). Among these, cholesterol polyps are the most common benign variety (constituting 78.5%), whereas adenomas only make up 2.2% of cases. Malignant polyps manifest as gallbladder carcinomas. The most prevalent type of mucoepidermoid carcinoma (MEC) in the gallbladder is associated with pyloric/pseudopyloric gland metaplasia, comprising a frequency of 66%–84%.

MEC within the gallbladder may encompass various types, including intestinal, pyloric, and mucous gland types, characterized by the presence of goblet and paneth cells. MEC can arise congenitally or as a secondary response to conditions such as cholecystitis, cholelithiasis, or tumor growth.^5^ Of note, intestinal‐type MEC, in particular, carries a risk of progressing into malignancy.^6^ Adenomas, constituting about 10% of gallbladder polyps detected via ultrasound, have been documented in approximately 0.3%–0.5% of gallbladders surgically excised for either cholelithiasis or chronic cholecystitis.^7^ Gallbladder adenomas manifest in four distinct cytological patterns: pyloric, intestinal, foveolar, and biliary.^4^ Typically asymptomatic, they may become problematic when multiple, large, or detached adenomas lead to the presence of free‐floating fragments within bile ducts or association with gallstones, thereby inducing symptomatic cholelithiasis.

Gallbladder cancer has a poor prognosis and is often incidentally detected during routine cholecystectomy due to its nonspecific presentation.^8^ Pyloric gland adenoma carries the risk of progressing into adenocarcinoma, with this malignant transition occurring in up to 47% of cases across all locations.^9^ Based on the examination of seven adenomas with malignant changes and the identification of adenomatous residue in 15 out of 79 (19%) invasive carcinomas in a study involving 1605 resected gallbladder specimens, Kozuka et al. proposed an adenoma‐carcinoma sequence.^10^ In another study involving 1847 cholecystectomy specimens, Lee et al. observed that malignant transformation took place in 23.5% of gallbladder adenomas.^11^ Gallbladder cancer was not suspected on preoperative imaging in the reported case. This concurred with the intraoperative findings and therefore, no formal lymphadenectomy was performed.

Recent research has showed the significance of CDX2 (homeobox domain–containing transcription factor) in not only intestinal inflammation but also in the development of tumors making CDX2 as a noteworthy marker for gallbladder adenoma.^12^, ^13^

Surgical resection of gallbladder polyps is recommended in patients with symptoms such as biliary‐type pain and dyspepsia. Intraoperative suspicious findings such as a mass, collaterals, cirrhosis and difficulty in completing the case laparoscopically may warrant conversion to open. Despite the intraoperative findings, we were able to complete the reported case laparoscopically. For asymptomatic individuals, resection is suggested^1^ if (I) they are over 50 years old; (II) their polyps are solitary and greater than 10 mm in size; (III) they have concurrent gallstones, or (IV) their polyps grow continuously on ultrasound exams. For polyps, 6–9 mm in diameter with no signs of malignancy, a repeat ultrasound in 6 months is recommended. If there are no significant changes, a new ultrasound in 12 months is advised; and if these exams are both stable, no further imaging studies are needed.^4^ A comprehensive literature review is summarized in Table 1.

**TABLE 1:** Literature review of reported cases of pyloric gland adenoma.

Author	Specimens examined	GB pyloric gland adenoma	Detached	High grade dypalsia/Malignancy
Nakanuma et al.^14^	104	7	No	NA
He et al.^15^	24	24	6	7
Yang et al.^16^	2	2	No	No
Chlumská et al.^17^	23	2	NA	NA
Nagata et al.^13^	58	58		2
Adsay et al.^2^	123	24		68
Köseoğlu et al.^18^	1	1	No	1
Vieth et al.^1^	90	2	NA	0
Wani^19^	29	29	NA	NA
Kushima et al.^20^	1	1		No
Albores‐Saavedra et al.^21^	201	165		44
Bronkhorst et al.^22^	1	1		No
Takei et al.^23^	63	59	No	20
Taskin et al.^24^	643	41	NA	NA
Itoi et al.^25^	252	68	NA	21
Rowan et al.^26^	19	15	NA	3
Summers et al.^27^	1	1		0
Yanagisawa et al.^28^	100	18	NA	0
Chang et al.^29^	248	19	0	0
Mohamed et al.	1	1	Yes	0

To conclude, a pyloric gland adenoma involving the gall bladder is a rare finding which may be missed and therefore should be considered as a differential diagnosis for any case of gall bladder polyp. Associated suspicious lymphadenopathy should be excised as in currently reported case which proved to be ectopic tissue. A thorough histopathological examination is necessary to establish the diagnosis. Due to the risk of evolving adenocarcinoma, surgical resection should be performed when possible and remains the mainstay of treatment. Identification of such cases earlier through a high index of suspicion can lead to better management and prognosis for the patient.

## AUTHOR CONTRIBUTIONS

**Mohamed H. Khalaf:** Methodology; writing – original draft. **Lina M. Mohamed:** Data curation; investigation; methodology; writing – original draft. **Raed M. Al‐Zoubi:** Methodology; writing – original draft. **Ahmad Zarour:** Conceptualization; data curation; investigation; methodology; writing – original draft. **Mohamed Said Ghali:** Conceptualization; data curation; investigation; methodology; writing – original draft.

## FUNDING INFORMATION

This research did not receive any specific grant from the public, commercial, or not‐for‐profit funding agencies.

## CONFLICT OF INTEREST STATEMENT

The authors of this manuscript have no conflicts of interest to declare. All co‐authors have seen and agree with the manuscript's contents and there is no financial interest to report.

## ETHICAL APPROVAL

Written informed consent was obtained from the patient for publication of this case report and any accompanying images. A copy of the written consent is available for review by the Editor‐in‐Chief of this journal. The Medical Research Center and Institutional Review Board (IRB) of Hamad Medical Corporation (HMC) confirmed the patient's consent, confirmed that data were anonymized and agreed with publication.

## CONSENT

Written informed consent was obtained from the patient to publish this report in accordance with the journal's patient consent policy.

## Data Availability

Data will be made available on request.
